# Profiling the urinary microbiome in men with calcium-based kidney stones

**DOI:** 10.1186/s12866-020-01734-6

**Published:** 2020-02-28

**Authors:** Jing Xie, Jian-sheng Huang, Xiang-jiang Huang, Jun-ming Peng, Zhou Yu, Ye-qing Yuan, Ke-feng Xiao, Ji-nan Guo

**Affiliations:** Department of Urology, Shenzhen People’s Hospital, The Second Clinical Medical College of Jinan University, Shenzhen, 518020 China

**Keywords:** Kidney stone, Calcium-based, Microbiome, Urine, Renal pelvis

## Abstract

**Background:**

The dogma that urine is sterile in healthy individuals has been overturned by recent studies applying molecular-based methods. Mounting evidences indicate that dysbiosis of the urinary microbiota is associated with several urological diseases. In this study, we aimed to investigate the urinary microbiome of male patients with calcium-based kidney stones and compare it with those of healthy individuals.

**Results:**

The diversity of the urinary microbiota in kidney stone patients was significantly lower than that of healthy controls based on the Shannon and Simpson index (*P* < 0.001 for both indices). The urinary microbiota structure also significantly differed between kidney stone patients and healthy controls (ANOSIM, R = 0.11, *P* < 0.001). Differential representation of inflammation associated bacteria (e.g., *Acinetobacter*) and several enriched functional pathways were identified in the urine of kidney stones patients. Meanwhile, we found the species diversity, overall composition of microbiota and predicted functional pathways were similar between bladder urine and renal pelvis urine in kidney stone patients.

**Conclusions:**

A marked dysbiosis of urinary microbiota in male patients with calcium-based kidney stones was observed, which may be helpful to interpret the association between bacteria and calcium-based kidney stones.

## Background

Nephrolithiasis is a common urological disorder worldwide, with a prevalence of 5–20% in different geographic regions and a recurrence rate of 50% at 10 years of follow-up [1]. The prevalence of nephrolithiasis has progressively risen during the last 30 years [2]. In China, the prevalence of nephrolithiasis in the periods of 1991–2000, 2001–2010, and 2011 to date was reported to be 5.95, 8.86 and 10.63%, respectively [3]. Similar increases in prevalence exist in a variety of systemic diseases, such as diabetes, cardiovascular disease and metabolic syndrome [4–6]. Moreover, a recent population-based study demonstrated that multiple classes of oral antibiotics exposure is associated with increased odds of nephrolithiasis [7]. Interestingly, human microbiome could be affected by all these factors, indicating its potential role in the pathophysiology of nephrolithiasis.

The term human microbiome is defined as all genetic materials of micro-organisms existing in different regions of the body. In the past few years, the role of gut microbiome on urine oxalate excretion and kidney stone formation has been a hot issue. Early study identified distinct gut microbiome and enrichment of oxalate metabolizing bacterial species in nephrolithiasis patients [8]. *Oxalobacter formigenes*, an oxalate degradation bacterium, was reported to be negatively associated with urinary stones and reduce urinary oxalate excretion when administered orally as a probiotic [9]. Despite the promising preliminary data, further studies showed contradictions as to the colonization rate of *O. formigenes*, ranging from 0 to 100% in kidney stone formers and 11–100% in individuals with no history of nephrolithiasis [10]. In addition, trials designed to degrade urinary oxalate with probiotics containing *O. formigenes* have been disappointing so far [11].

The urinary microbiome, identified in healthy individuals, is associated with several urologic diseases such as incontinence, genitourinary cancer and urinary tract infection [12, 13]. Early research observed that patients with non-struvite kidney stones often had positive urine cultures, indicating urinary microorganisms are associated with almost all types of kidney stones [14]. In a recent study, the urinary microbiome was showed to hold more relevant for urinary stones than the gut microbiome [15]. Collectively, these results suggest that urinary microbiome may be closely associated with nephrolithiasis. However, there have been limited studies to date that evaluate the association between urinary microbiome and calcium-based kidney stones [16]. In addition, whether the flora of bladder urine is distinct from that of renal pelvis urine also remains equivocal.

In the present study, we utilized 16S rRNA gene sequencing to characterize the urinary microbiome potentially associated with calcium-based kidney stones. The aim of our research was to (1) determine if the microbiome of bladder urine is significantly different between kidney stone formers and healthy individuals; (2) determine if the microbiome of bladder urine is distinct from that of renal pelvis urine in nephrolithiasis patients. (3) predict functional pathways that significantly enriched in the urinary microbiome of kidney stone formers.

## Results

### General characteristics of kidney stone patients and controls

Urine samples were collected from a total of 43 subjects, and the demographic and clinical data was listed in Table 1. Age, gender, and body mass index showed no significant difference between kidney stone patients and healthy controls. Although comorbidities such as hypertension, diabetes and coronary artery disease were more common in kidney stone formers, they all did not reach statistical significance. The majority of renal stone patients were first onset (20/22, 90.9%) and only two patients were recurrent. All kidney stones were primarily calcium-based and composed of calcium oxalate, calcium phosphate, or a mixture of components. Pure calcium phosphate, uric acid, cystine or struvite stones were not identified. Antibiotics were given immediately after sample collection and no associated postoperative infections were identified in this study.

**Table 1:** Demographic and clinical data for the kidney stone formers and healthy subjects

	Stone formers (***n*** = 22)	Healthy controls (***n*** = 21)	***P*** value
Age, years	46.9 ± 10.1	44.2 ± 12.1	0.435
Female	0	0	1.0
BMI, kg/m^2^	24.3 ± 3.28	24.4 ± 2.57	0.876
Comorbidities			
Hypertension	5	2	0.410
Diabetes	2	0	0.488
Coronary artery disease	1	0	1.0
Stone composition			NA
CaOx	18	NA	
CaOx + CaPhos	3	NA	
CaPhos	0	NA	
CaOx + Uric acid	1	NA	
Uric acid	0	NA	

*BMI* Body mass index, *CaOx* Calcium oxalate, *CaPhos* Calcium phosphate

### Sequencing data and biodiversity of the urine microbiome

**Fig. 1:**
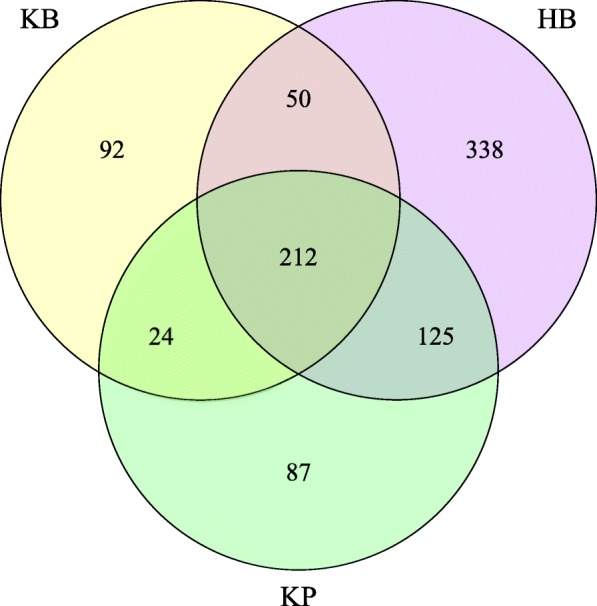
Venn diagram of overlapping OTUs. A total of 928 OTUs were detected with 338 OTUs in HB samples only, 92 OTUs in KB samples only, 87 OTUs in KP samples only and 212 OTUs in all urine samples

In total, 5,906,796 clean reads were obtained from the 65 urine samples. The median number of reads in kidney stone patients was 94,966 and in healthy controls was 126,090. The reads were classified into 928 unique operational taxonomic units (OTUs) at 97% similarity level that were used for downstream analysis. We defined three groups according to the kidney stone status and specimen-type: HB represents bladder urine collected from healthy controls, KB represents bladder urine from kidney stone patients, while KP represents renal pelvis urine from kidney stone patients. The HB group showed the largest amount of OTUs, and there was substantial overlap in the OTUs composition among HB, KB and KP groups (Fig. 1). Significant more OTUs were identified in the urine of healthy controls, with an average of 96 OTUs in HB group and 60 OTUs in KB group (*P* = 0.046).

**Fig. 2:**
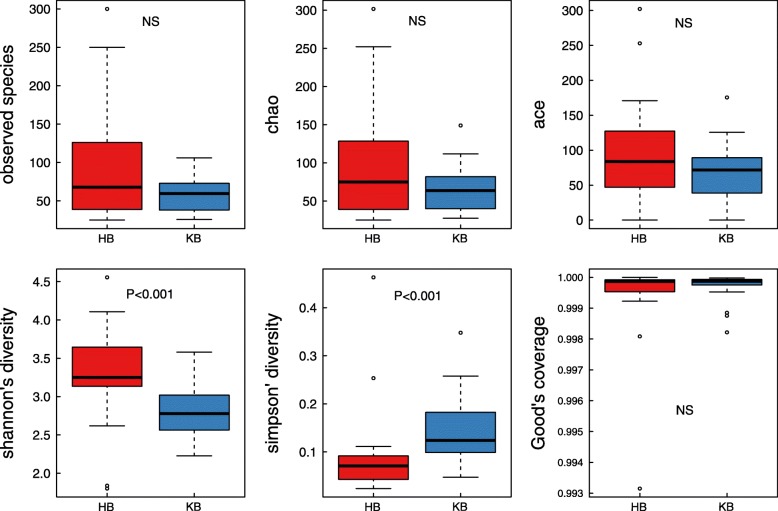
Microbial α − diversity of urine samples. The α − diversity indices include observed species index, Chao 1 index, Ace index, Shannon index, Simpson index and Good’s coverage index. Shannon diversity index and Simpson’s diversity index were significantly different between HB and KB group

**Fig. 3:**
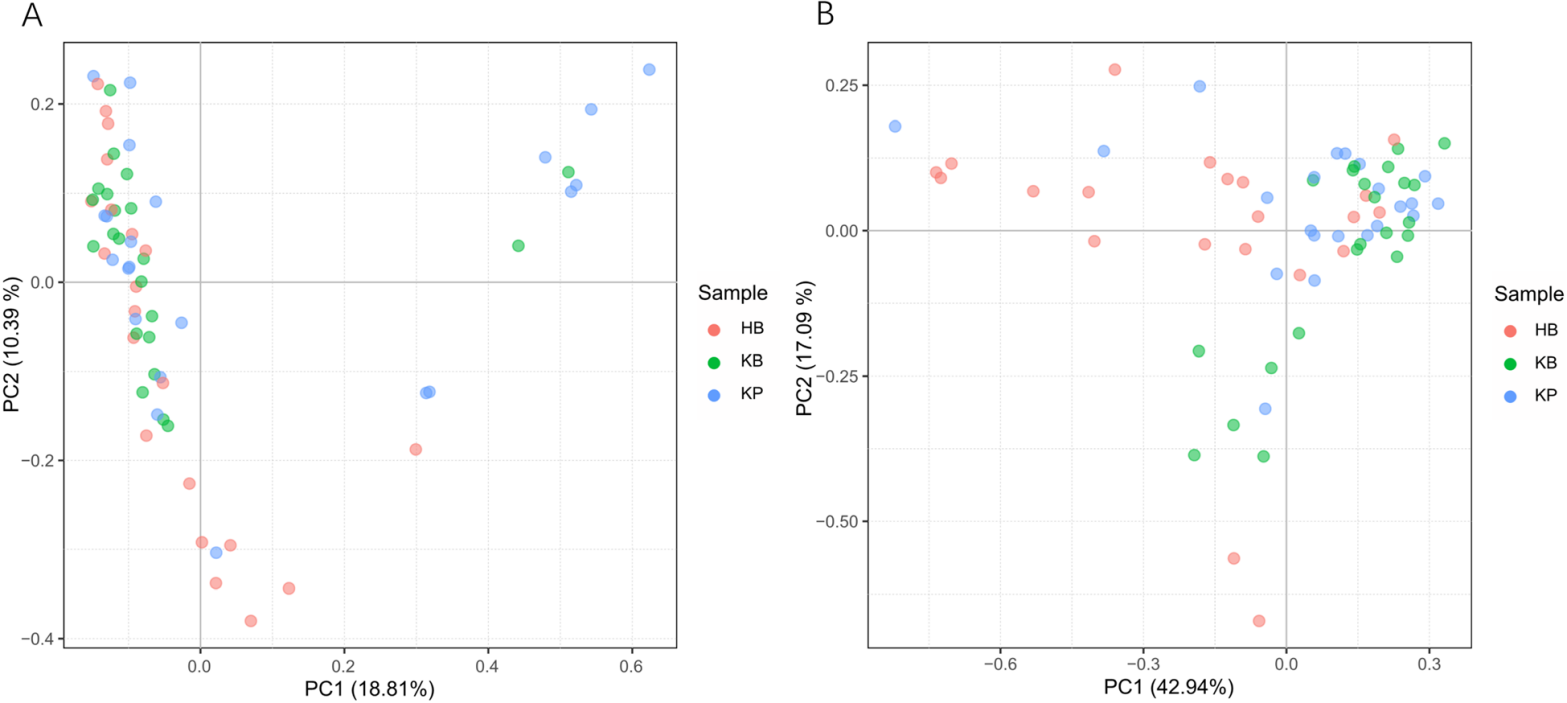
Microbial β − diversity analysis. PCoA plot of unweighted (**a**) and weighted (**b**) UniFrac metrics for HB (red dots), KB (green dots) and KP (blue dots) groups. Samples from KB and KP groups clustered closer in proximity to each other than HB samples

For α − diversity, the values of Good’s coverage index of all libraries were above 99%. The α − diversity indices, including observed species, chao 1 index, ACE index, Shannon diversity index, of the microbiota in HB group were all higher than those of KB group (Fig. 2). Moreover, significant differences were observed in Shannon diversity index and Simpson’s diversity index between HB and KB groups (*P* < 0.001 for both indices). The α − diversity of urinary microbiota between KB and KP group was also evaluated, and all indices showed no significant difference. For β − diversity, we applied unweighted and weighted principal coordinate analysis (PCoA) to display discrepancy among the three groups. It showed that KB and KP samples clustered closer in proximity to each other than HB samples (Fig. 3). We further performed analysis of similarities (ANOSIM), and found the urinary microbiota structure was significantly different between KB and HB groups (ANOSIM, R = 0.11, *P* < 0.001), while the microbiota structure between KB and KP groups was similar (ANOSIM, R = 0.008, *P* = 0.251).

### Taxonomic analysis of urine microbiota composition

To identify the differentially represented taxa in kidney stone patients and controls, we compared the relative abundance of microbiota between KB and HB group at different taxonomic levels. At phylum level, a statistically significant difference was observed between these two groups in the average abundance of Bacteroidetes, Proteobacteria and Firmicutes. Namely, KB group showed a higher average representation of Proteobacteria (51.8% vs 36.6%, *p* = 0.01) and a lower average representation of Firmicutes (29.3% vs 36.1%, *p* = 0.02) and Bacteroidetes (6.4% vs 19.4%, *p* < 0.001). Significant abundance differences of numerous taxa were also noted between KB and HB groups at other taxonomic levels (Table 2). The relative abundance of *Faecalibacterium* and *Lactobacillus* was also lower in KP and KB groups compared to HB group, although not statistically significant.

**Table 2:** Comparison of average relative abundance of bladder urine microbiome in kidney stone patients and healthy subjects at different taxonomic levels

Taxa		Average abundance (%)			Prevalence (%)	
		***P*** value	HB	KB	HB	KB
Phylum	*Bacteroidetes*	< 0.001	19.362	6.402	100	100
	*Proteobacteria*	0.013	36.641	51.797	100	100
	*Firmicutes*	0.024	36.114	29.318	100	100
Family	*Moraxellaceae*	< 0.001	11.960	32.655	100	100
	*Prevotellaceae*	0.001	8.992	1.572	100	73
	*Odoribacteraceae*	0.008	0.110	0.000	29	0
	*Fusobacteriaceae*	0.012	0.374	1.303	43	73
	*Porphyromonadaceae*	0.014	0.754	0.341	67	27
	*Enterococcaceae*	0.041	0.494	0.913	57	73
	*Planococcaceae*	0.047	0.132	0.487	38	59
Genus	*Acinetobacter*	< 0.001	10.996	31.383	100	100
	*Prevotella*	0.001	9.377	1.628	100	73
	*Desulfovibrio*	0.008	0.178	0.000	29	0
	*Eubacterium*	0.008	0.039	0.000	29	0
	*Odoribacter*	0.008	0.083	0.000	29	0
	*Fusobacterium*	0.012	0.374	1.297	43	73
	*Parabacteroides*	0.017	0.540	0.252	57	23
	*Lysinibacillus*	0.020	0.069	0.468	29	55
	*Oscillospira*	0.039	0.626	0.019	38	14
Species	*Acinetobacter johnsonii*	< 0.001	7.310	26.039	100	100
	*Prevotella copri*	< 0.001	7.272	0.431	86	59
	*Prevotella stercorea*	0.002	0.422	0.031	57	14
	*Clostridium sartagoforme*	0.004	0.099	0.000	33	0
	*Bacteroides barnesiae*	0.008	0.369	0.000	29	0
	*Eubacterium biforme*	0.008	0.031	0.000	29	0
	*Lysinibacillus boronitolerans*	0.020	0.069	0.468	29	55
	*Bacteroides ovatus*	0.021	1.084	0.212	57	23
	*Parabacteroides distasonis*	0.030	0.337	0.047	43	14
	*Bacteroides fragilis*	0.033	0.368	0.070	43	14
	*Bacteroides plebeius*	0.037	1.473	1.876	71	91
	*Veillonella parvula*	0.047	0.364	0.213	38	9

**Fig. 4:**
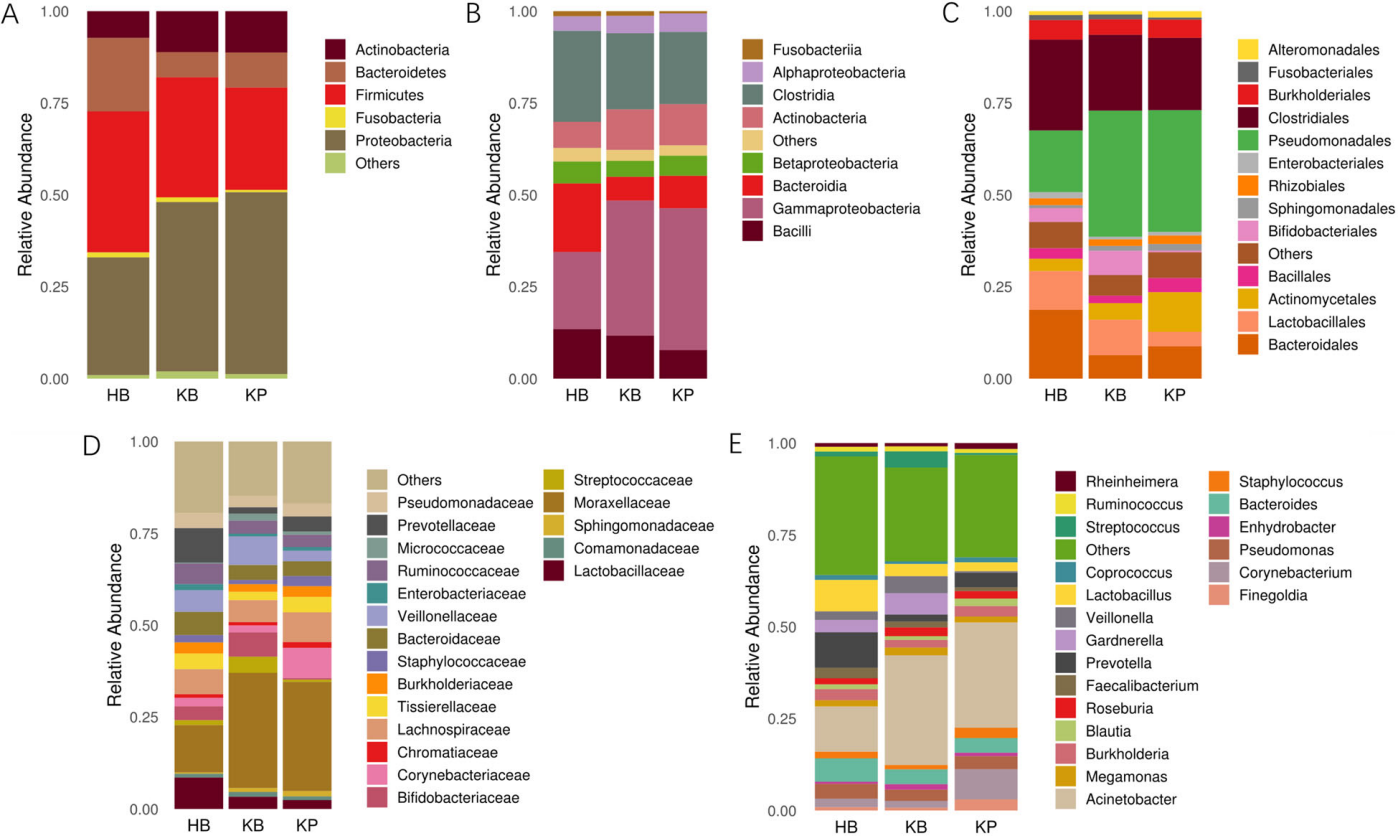
Bacterial average relative abundance in HB, KB and KP groups at different taxonomic levels. **a** phylum, **b** class, **c** order, **d** family, **e** genus. Average distribution of major taxa is represented by bar graphs. Unclassified genera or genera with a relative abundance < 1% are grouped as “Other”

Of interest, we also analyzed the microbiota of paired bladder urine and renal pelvis urine collected from kidney stone patients. At phylum or class level, the overall bacterial compositions of KB and KP groups were quite similar (Fig. 4a-b). However, there were a few taxa differentially represented in these two groups at other taxonomic levels (Fig. 4c-e). A higher average representation of *Anoxybacillus* (1.2% vs 0.2%, *p* = 0.01) and lower average representation of *Fusobacterium* (0.6% vs 1.3%, *p* = 0.02) was observed in KP group at genus level.

### Specific urinary genera associated with kidney stones

**Fig. 5:**
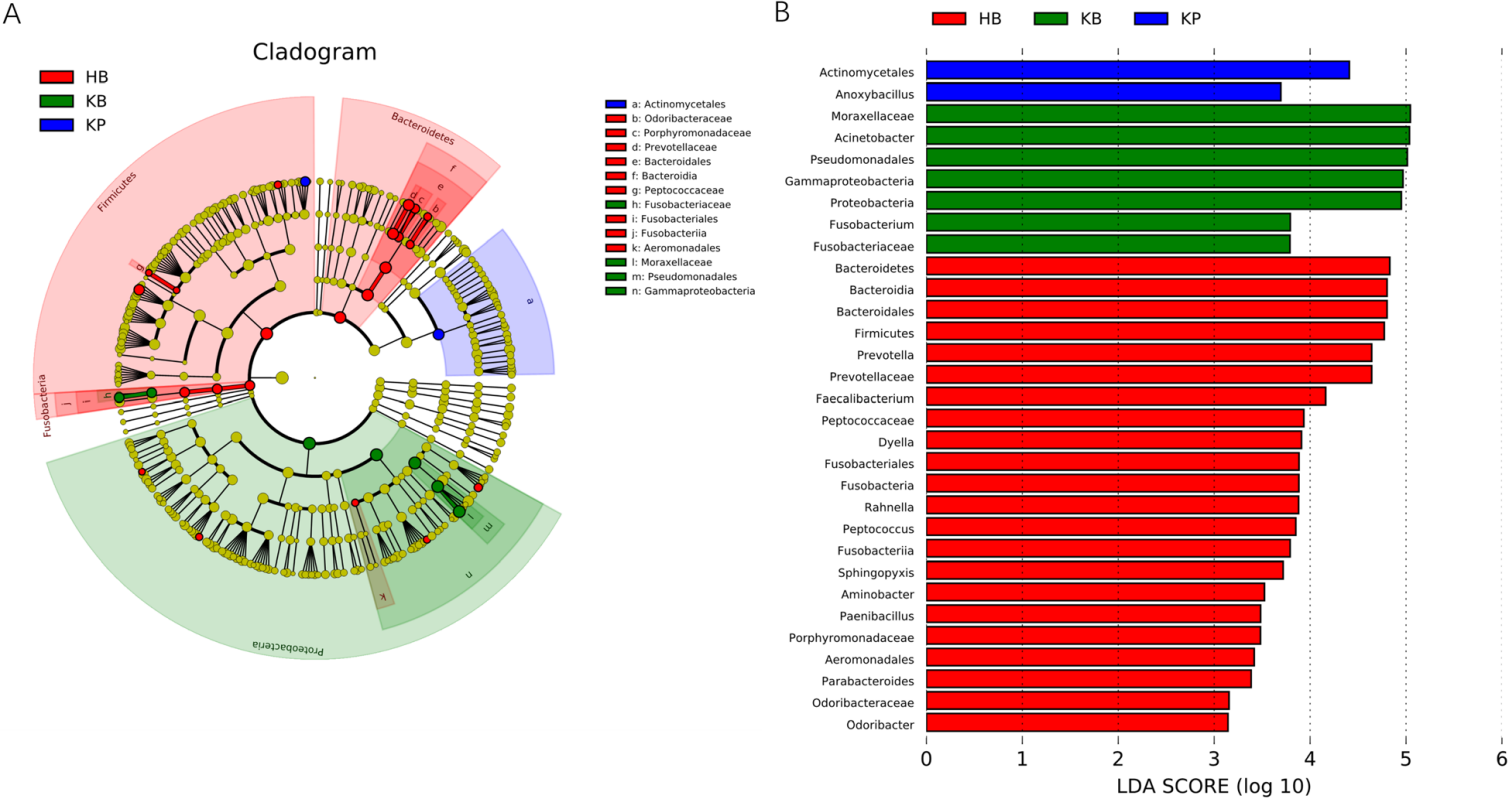
Cladogram (**a**) and LEfSe analyses (**b**) of microbiomes among HB (red), KB (green) and KP (blue) groups. Taxa in graph were with LDA score threshold > 2.0 and statistically significant (*p* < 0.05)

To confirm the differentially abundant taxa in kidney stone patients and controls, we further applied LEfSe, a software using algorithm for high-dimensional biomarker discovery. Only taxa with logarithmic linear discriminant analysis (LDA) score more than 2.0 and *P* < 0.05 in Wilcoxon test were considered differentially represented. LEfSe identified 31 discriminative features with significant different relative abundance among HB, KB and KP groups (Fig. 5). The taxa at genus level that differentiated the three groups most were *Prevotella* in HB group, *Acinetobacter* in KB group and *Anoxybacillus* in KP group.

### Potential functional pathways associated with kidney stone

Having observed a distinct urinary microbiota in kidney stone patients, we further evaluated whether the different bacterial community was associated with specific alterations involved in metabolic processes. The functional pathways of urinary microbiome in HB, KB and KP samples were inferred using PICRUSt tool. Compared to HB group, the significantly enriched KEGG pathways in KB groups included proximal tubule bicarbonate reclamation, ion channels, linoleic acid metabolism and renin−angiotensin system (Supplementary Figure. 1). Meanwhile, the predicted KEGG pathways showed no significant difference between KB and KP groups (Supplementary Figure. 2).

## Discussion

In the present study, we utilized 16S rRNA gene sequencing to explore the urinary microbiome in male calcium-based kidney stone formers and age-matched healthy individuals. The noted improvement in our study was the strict inclusion criteria, aiming to control various confounding factors. Our results showed significantly reduced species diversity and altered microbial profile in the urine of kidney stone patients compared to controls. Several differentially represented taxa and functional pathways were found in HB and KB groups. In addition, we found the overall bacterial composition and predicted functional pathways of bladder urine was similar to that of renal pelvis urine in kidney stone patients.

Urolithiasis is a common urological disorder with diverse pathologies and the factors contributing to the increasing prevalence are currently unknown. In the previous literature, the mechanisms that urea-producing bacteria promoting struvite stone formation have been well documented. However, the potential mechanism that bacteria contribute to calcium-based stone, the vast majority of kidney stones, remains obscure. One hypothesis is that bacteria adhere to crystal and promote its growth and aggregation. This is supported by findings that bacteria such as *Enterobacteriaceae* selectively aggregated to oxalate calcium crystal and increased the number of aggregations [17]. Similar crystal aggregation ability was observed in *Staphylococcus* and *Streptococcus* species in vitro [18]. Another possibility is that bacteria may alter urine supersaturation via production of citrate lyase, which decreases the urine citrate levels and lead to crystal formation [19]. Lastly, bacteria may induce an inflammatory response and the release of proinflammatory proteins, which form the stone matrix inner core and progress from crystal to stone [20].

The main finding of this study was that we demonstrated distinct urinary microbiota in kidney stone patients compared to healthy subjects. Our results showed that nephrolithiasis patients had significant lower species diversity in urine. According to previous literature, decreased microbiota diversity was related to inflammation and implicated in diseases such as obesity and type II diabetes [21]. Moreover, we found several bacterial taxa associated with inflammation were overrepresented or underrepresented in the urine of kidney stone patients. The most differentially represented taxa at genus level were *Acinetobacter* in kidney stone patients, and *Prevotella* in healthy controls. As opportunistic pathogens, *Acinetobacter* are associated with urinary tract infection in individuals with underlying medical risk factors, such as diabetes mellitus and immunosuppression [22]. Interestingly, the abundance of *Acinetobacter* was showed to be higher in the faeces of nephrolithiasis patients and the urine of bladder cancer patients compared to controls, although its association with plasma trace elements or bladder cancer recurrence/progression had not been identified [23, 24]. *Prevotella* are classically considered as commensal bacteria and known to colonize the gastrointestinal tract, vaginal tract and urinary tract. It could synthetize short-chain fatty acids, which were able to protect against inflammation in acute kidney injury [25]. The decreased level of *Prevotella* favours inflammatory processes and has been implicated in several pathological conditions, including type 2 diabetes, diabetic nephropathy and chronic prostatitis [26, 27].

In summary, our results revealed significantly decreased species diversity, enrichments of proinflammatory bacteria and underrepresentation of anti-inflammatory taxa in the urinary microbiota of kidney stone patients. Similar trends were showed by Zampini and colleagues that a long-term shift in urinary tract microbiome may increase the risk for urinary stones, although not excluding subjects using antibiotics [15]. We also predicted several functional pathways which were significantly enriched in the urinary microbiome of kidney stone patients compared to healthy controls. Among these pathways, ion channels are key regulators of the cell membrane and have been demonstrated as an entrance gate in bacteria-host interactions [28]. In an infection-induced urolithiasis rat model, the activities of calcium related ion channels were reported to be influenced by bacterial infection, and correlated with chronic inflammation of the kidney along with rapid aggregation of stones [29]. Moreover, transient receptor potential vanilloid 5 (TRPV5), a member of the transient receptor potential family of ion channels, has also been proved to be closely associated with urinary stone formation [30]. In the present study, our results revealed enriched ion channels pathway in the urine of nephrolithiasis patients, but its specific role in kidney stone formation still remains unclear and needs further investigation. Nevertheless, it is reasonable to speculate that bacteria might influence the formation of calcium-based stones via modulation of inflammatory processes.

Another important finding in this study was the similarity of overall bacterial composition between bladder urine and renal pelvis urine in kidney stone patients. Traditionally, bacteria are considered to access the upper urinary tract under certain conditions, such as urinary reflux or bacteria translocation in severe systemic disease. However, a preliminary study showed bacteria could be detected in the upper tract urine of kidney stone patients without urinary tract infections [17]. Due to the small sample size, the author did not compare the microbiota between bladder urine and upper tract urine. In a recent study, Dornbier and colleagues found that there was no significant difference in the microbial composition of bladder urine and upper tract urine in urinary stone patients [16]. It is worth noting that ureteral stents were placed in the majority of patients (50/52, 96.1%) in that study, which may potentially influence the urinary microbiota. In the present study, we found that the species diversity and overall composition of microbiota was similar between KB and KP groups, after excluding confounding factors such as antibiotic use and ureteral stent placement. In addition, our PICRUSt results showed no significant difference with regard to the predicted functional pathways between KB and KP groups. Meanwhile, we also noted that there were a few taxa (e.g., *Anoxybacillus*) differentially represented in KP group, remaining an area for future research.

Some limitations should be noted when interpreting our results. First, all participants were Chinese and the sample size is relatively small, limiting generalizability and comparison of stone subtypes. Further largescale studies are necessary to investigate the urinary tract microbiota across ethnicity and stone type. Second, this study did not include female subjects, mainly due to their lower morbidity of kidney stones and higher positive rate of urine routine tests. In the future, we will conduct a more comprehensive research after recruiting adequate females in line with our inclusion criteria. Additionally, the association of risk factors for lithogenesis in urine and urine microbiota was not evaluated, because the vast majority of kidney stone patients were first onset and 24 h urine analyses were not performed. Finally, like most metagenomic studies, we cannot comment as to whether altered urinary microbiota in kidney stone patients was a contributor or the result of kidney stone formation. All these questions will certainly be the focus of future research.

## Conclusions

In conclusion, our study revealed distinct urinary microbiota in male kidney stone patients compared to healthy individuals, and similar microbiota between bladder urine and renal pelvis urine. Several predicted functional pathways and bacteria associated with inflammation were found to differentially represent in the urinary tract of kidney stone patients. We speculated that bacteria might influence the formation of calcium-based kidney stones via modulation of inflammatory processes. Our findings may provide useful information to interpret the association between bacteria and calcium-based kidney stones.

## Methods

### Recruitment of participants

We recruited a total of 43 adult males, including 22 kidney stone formers and 21 age-matched healthy volunteers at Shenzhen People’s Hospital. All nephrolithiasis patients were diagnosed by ultrasonography, abdominal plain film, intravenous pyelography or computed tomography, and kidney stones were confirmed during endoscopic surgery. The chemical composition of surgically removed stones was analyzed by infrared spectroscopy.

In order to control the confounding factors that might affect urinary microbiome, we set strict exclusion criteria. For healthy controls, exclusion criteria included personal history of urinary stones, episodes of renal colic or imaging confirmed urinary stones. All healthy controls underwent ultrasonography to confirm the lack of asymptomatic renal calculus. For kidney stone patients, exclusion criteria included struvite stones, concurrent ureteral calculus, moderate to severe hydronephrosis, and ureteral stent or catheter placement before sample collection. Excluded from both groups were subjects using antibiotic within 30 days, with urinary tract infections or positive urine culture, congenital abnormalities of the urinary tract, history of major urological surgery, diabetes with poorly controlled glucose, autoimmune disease, chronic kidney disease with blood creatinine > 1.4 mg/dL and age (< 20 years or > 70 years old).

### Sample collection and processing

Bladder urine samples were obtained by transurethral catheterization from all participants. For nephrolithiasis patients, paired renal pelvis urine samples were collected on the side of kidney stones via ureteral catheter using aseptic technique, prior to surgery or ureteral stent insertion. Before the renal pelvis urine collection, the bladder was voided by catheter with an attempt to control the mixture of bladder urine. All samples were collected prior to antibiotic use and stored in sterile containers at − 80 °C within 1 h from collection. The volume of each urine sample was approximately 12 ml and the time for renal pelvis urine collection was approximately 10 min.

### DNA extraction and 16S rRNA amplicon sequencing

Prior to DNA extraction, all samples were centrifuged 12,000 g for 10 min at 4 °C. Pellets were re-suspended and mixed with DNA-free phosphate buffered saline. Genomic DNA was extracted from all samples using DNeasy PowerWater Kit (MoBio, USA). Integrity of DNA was verified with agarose gel electrophoresis and the DNA concentration was quantified by Qubit® 2.0 Fluorometer (Life Technologies, USA). All DNA extractions were stored at − 20 °C until further processing.

The V3-V4 region of the 16S rRNA gene was amplified by polymerase chain reaction with primers shown as follows: V3-V4-341F: 5′- CCTACGGGNGGCWGCAG-3′ and 907R: 5′-TACNVGGGTATCTAATCC-3′. Polymerase chain reaction was performed using the following conditions: 3 min denaturation at 98 °C; 30 cycles of denaturation at 98 °C for 45 s, annealing at 55 °C for 45 s, elongation at 72 °C for 45 s; and final extension at 72 °C for 7 min. The amplicons were purified by the AMPure beads (Axygen, USA), and barcoded libraries were sequenced on the Illumina Hiseq2500 platform. Sterile phosphate buffered saline with and without bullet blender beads were used as negative controls during processing.

### Bioinformatic analysis

Raw sequencing data was pre-processed to eliminate low-quality reads and adapter pollution by using Mothur [31]. Clean reads were merged to tags using FLASH software [32] and the latter were assigned to OTUs based on 97% sequence similarity using USEARCH [33]. The representative sequences of each OTU were taxonomically classified by RDP Classifier [34] based on the Greengenes database [35]. Sequences associated with chimeras, chloroplasts and mitochondria were removed prior to downstream analyses [36]. Due to the low biomass nature of urine samples, the threshold for sequence positivity was conservatively set at a cutoff of 2000 sequence reads [16].

For α − diversity, observed species, chao 1 index, ACE index, Shannon index, Simpson index and Good-coverage index were calculated by Mothur. For β − diversity, both unweighted and weighted UniFrac distances were conducted using QIIME and shown by the PCoA [37]. Analysis was performed to find biomarkers differentially represented among the sample groups by LEfSe software [38]. The threshold on the logarithmic LDA score for discriminative features was 2.0. The functional pathways of bacterial community were inferred by utilizing PICRUSt algorithm [39]. In brief, the OTU table was imported to PICRUSt software and functional predictions were performed using Kyoto Encyclopedia of Genes and Genomes (KEGG) orthology.

### Statistical analyses

Statistical analyses were performed with the SPSS (version 21.0) and R software (version 3.4.1), considering *p* values < 0.05 as statistically significant. Clinical categorical variables were compared using Pearson’s chi-square test or Fisher’s Exact Test, while continuous variables were analyzed via a student’s t test. Age and body mass index were expressed as mean ± standard deviation. For α-diversity and taxonomic analysis, Wilcoxon rank-sum test or Kruskal-Wallis test were performed with R software. For β − diversity, statistical comparisons of weighted UniFrac distances were conducted by ANOSIM using the vegan package of R software.

## Supplementary information

**Additional file 1: Figure S1.** Microbial pathways that were significantly differentially enriched between HB and KB groups.

**Additional file 2: Figure S2.** Predicted microbial pathways were not significantly differentially represented between KB and KP groups.

## Data Availability

All data generated or analysed during this study are included in this published article and its supplementary information files. The datasets used and/or analysed during the current study are also available from the corresponding author on reasonable request.

## Abbreviations

ANOSIM: Analysis of similarities
HB: Bladder urine collected from healthy individuals
KB: Bladder urine collected from nephrolithiasis patients
KP: Renal pelvis urine collected from nephrolithiasis patients
LDA: Linear discriminant analysis
OTU: Operational taxonomic unit
PCoA: Principal coordinate analysis
