# Supplementary figures and images for: Profiling the urinary microbiome in men with calcium-based kidney stones
Source: BMC Microbiol. 2020 Feb 28;20:41. doi: 10.1186/s12866-020-01734-6 (PMC7049185; doi:10.1186/s12866-020-01734-6)

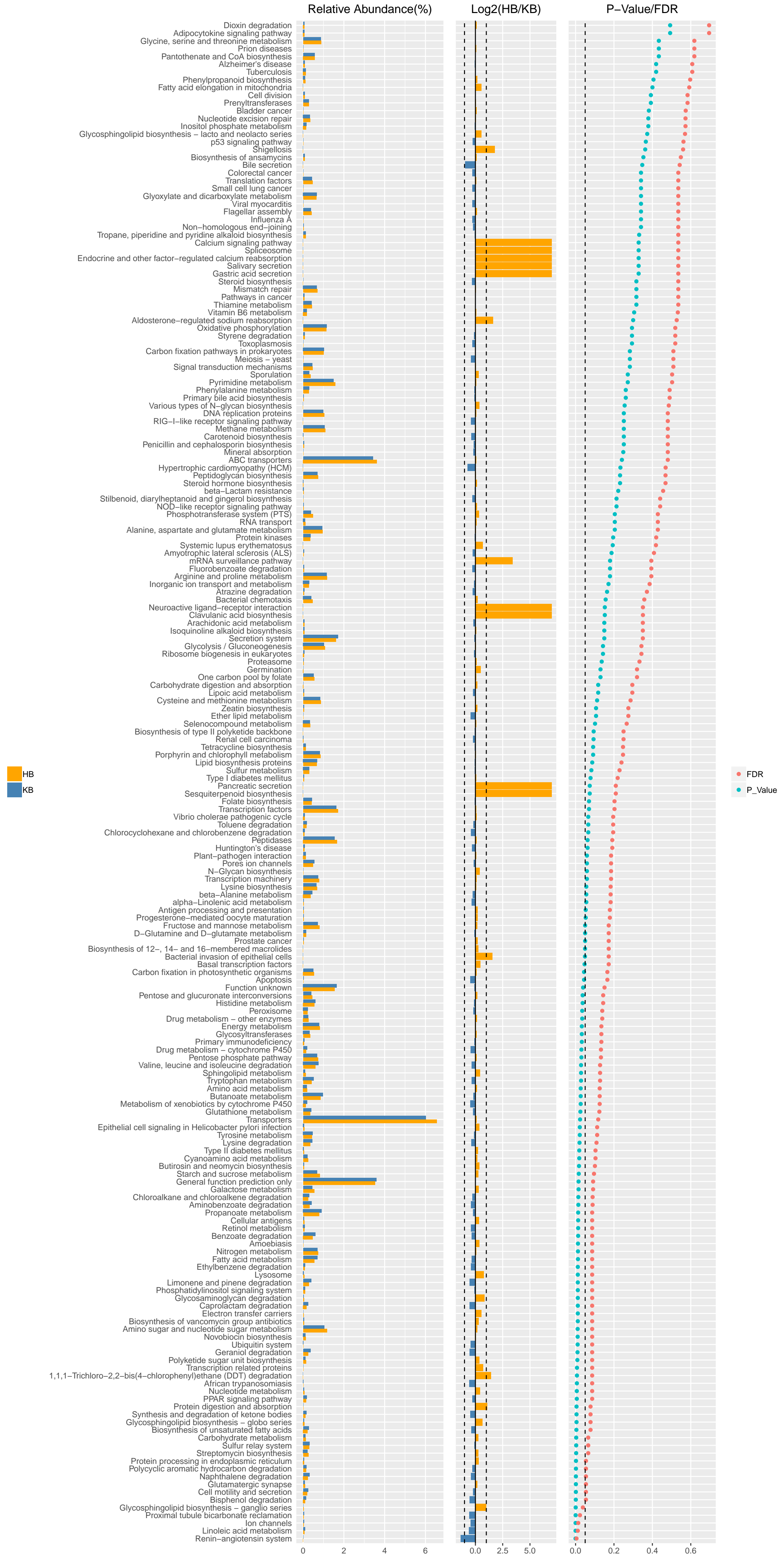

Supplement: Supplementary file 1 — Additional file 1: Figure S1. Microbial pathways that were significantly differentially enriched between HB and KB groups. [file 12866_2020_1734_MOESM1_ESM.pdf]

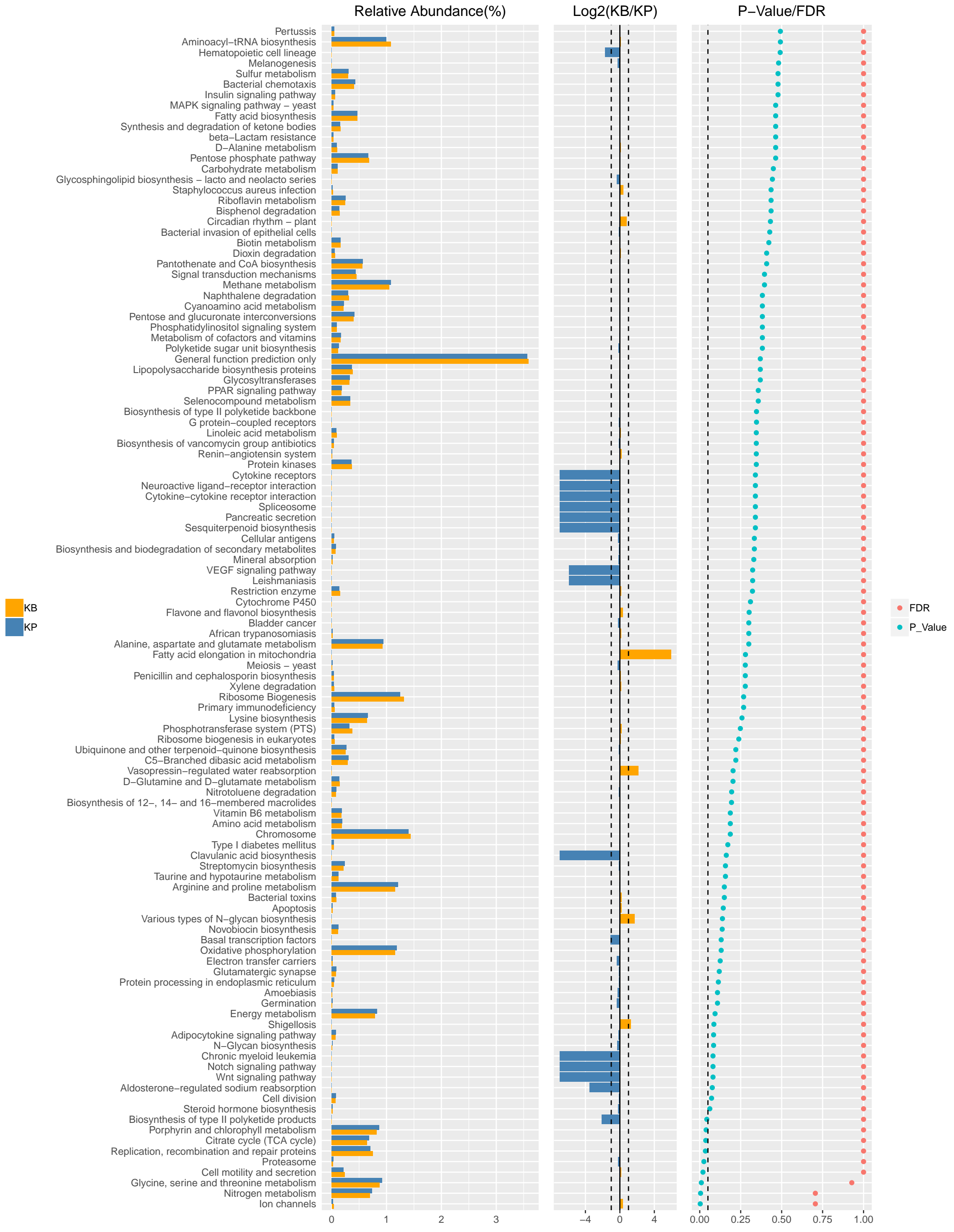

Supplement: Supplementary file 2 — Additional file 2: Figure S2. Predicted microbial pathways were not significantly differentially represented between KB and KP groups. [file 12866_2020_1734_MOESM2_ESM.pdf]
